# Comprehensive profiling and localisation of the matrix metalloproteinases in urothelial carcinoma

**DOI:** 10.1038/sj.bjc.6602931

**Published:** 2006-02-07

**Authors:** M J Wallard, C J Pennington, A Veerakumarasivam, G Burtt, I G Mills, A Warren, H Y Leung, G Murphy, D R Edwards, D E Neal, J D Kelly

**Affiliations:** 1 Department of Oncology, Hutchison MRC Research Centre, University of Cambridge, Hills Road, Cambridge CB2 2XZ, UK; 2 School of Biological Sciences, University of East Anglia, Norwich NR4 7TJ, UK; 3 Department of Pathology, Addenbrooke's Hospital, Cambridge CB2 2QQ, UK; 4 Department of Urology, Freeman Hospital, Newcastle NE7 7DN, UK

**Keywords:** MMP, urothelial cancer, laser capture microdissection, quantitative RT–PCR

## Abstract

The matrix metalloproteinases (MMPs) are endopeptidases which break down the extracellular matrix and regulate cytokine and growth factor activity. Several MMPs have been implicated in the promotion of invasion and metastasis in a broad range of tumours including urothelial carcinoma. In this study, RNA from 132 normal bladder and urothelial carcinoma specimens was profiled for each of the 24 human MMPs, the four endogenous tissue inhibitors of MMPs (TIMPs) and several key growth factors and their receptors using quantitative real time RT–PCR. Laser capture microdissection (LCM) of RNA from 22 tumour and 11 normal frozen sections was performed allowing accurate RNA extraction from either stromal or epithelial compartments. This study confirms the over expression in bladder tumour tissue of well-documented MMPs and highlights a range of MMPs which have not previously been implicated in the development of urothelial cancer. In summary, MMP-2, MT1-MMP and the previously unreported MMP-28 were very highly expressed in tumour samples while MMPs 1, 7, 9, 11, 15, 19 and 23 were highly expressed. There was a significant positive correlation between transcript expression and tumour grade for MMPs 1, 2, 8, 10, 11, 12, 13, 14, 15 and 28 (*P*<0.001). At the same confidence interval, TIMP-1 and TIMP-3 also correlated with increasing tumour grade. LCM revealed that most highly expressed MMPs are located primarily within the stromal compartment except MMP-13 which localised to the epithelial compartment. This work forms the basis for further functional studies, which will help to confirm the MMPs as potential diagnostic and therapeutic targets in early bladder cancer.

Over 60 240 new cases of urothelial carcinoma were diagnosed in the United States in 2004, and bladder cancer is now the fourth most common malignancy in males (Jemal *et al*, 2004). In the United Kingdom, cancer of the urinary bladder accounted for almost 5000 deaths in 2004. It is the fourth most common cancer in men and the eighth most common in women (CRUK Cancer Statistics, 2004).

Although the mortality associated with bladder cancer is naturally of concern, most patients present with noninvasive forms of the disease and with current treatment regimes, do not die as a direct result of their malignancy. Over 75% of patients present with one or more superficial tumours, and two thirds of these will develop recurrent disease (Lutzeyer *et al*, 1982), with 10–20% progressing to an invasive phenotype (Torti and Lum, 1984). The outcome for patients with invasive disease at presentation remains poor, with distant metastasis occurring in over 50% within 2 years and an average 5-year survival of only 50% (Raghavan *et al*, 1990). The early identification of those tumours likely to invade locally or to metastasize to distant sites is key to optimising the diagnosis and treatment of urothelial carcinoma.

The capacity to degrade and break down the basement membrane and extracellular matrix is essential for primary tumour cells to invade locally and spread to distant sites (Stamenkovic, 2000; Egeblad and Werb, 2002). The matrix metalloproteinases (MMPs) comprise a family of 24 extracellular zinc dependent endopeptidases with a broad spectrum of enzymatic activity against all components of the extracellular matrix (ECM). There are four primary endogenous tissue inhibitors of the MMPs (TIMPs). The physiological functions of the MMPs and TIMPs include organogenesis, tissue repair and wound healing (Vihinen and Kahari, 2002).

The patho-physiological roles of MMPs are, however, not limited to direct degradation and remodelling of the ECM; they are involved in angiogenesis, cell migration and proliferation, modulation of the host immune response and in the regulation of apoptosis (Chang and Werb, 2001). It is, therefore, understandable that members of the MMP family contribute to a number of disease processes including arthritis, atherosclerosis and cancer (Birkedal-Hansen *et al*, 1993). The evidence implicating MMPs in the progression and metastasis of visceral malignant disease is extensive and compelling. In urothelial carcinoma several of the well characterised MMPs, including MMP-2, MMP-9 and MT1-MMP (MMP-14), demonstrate increased expression and activity (Raghavan *et al*, 1990; Davies *et al*, 1993; Kanda *et al*, 2000; Hara *et al*, 2001).

It is evident that MMPs and TIMPs interact in a complex manner to regulate the extracellular microenvironment. Collectively, the MMP family can degrade all components of the extracellular matrix and there is significant overlap of substrate specificity between each MMP (Sternlicht and Werb, 2001; Egeblad and Werb, 2002). Many well-established MMPs have a broad proteolytic activity; however, not all MMPs share this property – recently isolated members such as MMP-28 appear to have only a single identified substrate, and for others the substrate or signalling pathway is unknown (Lohi *et al*, 2001).

While most MMPs are secreted, six membrane type MMPs (MT-MMPs) are bound to the cell surface through a C terminal sequence acting as either a type 1 transmembrane domain or as a signal for GPI anchoring (Seiki, 2002). The membrane-bound MMPs act on a broad spectrum of ECM substrates, and are also involved in the activation of other secreted MMPs and tumourigenic factors. MT1-MMP, in a complex with TIMP-2, converts pro-MMP-2 to the active form. With the probable exception of MT4-MMP, the other membrane bound MMPs show similar activity (Murphy *et al*, 1999). MT1-MMP is also responsible for the activation of proMMP-13 either directly or via MMP-2 activation (Knauper *et al*, 1997).

Consequently, some MMPs have been proposed as prognostic markers and therapeutic targets (Vihinen and Kahari, 2002), though phase III clinical trials with MMP inhibitors in advanced disease have shown disappointing results (Coussens *et al*, 2002). We postulate that accurate targeting of appropriate therapy to early, high-risk tumours may demonstrate significant benefits.

This present study uses an established and well-validated quantitative real time PCR assay to conduct a comprehensive profile of the entire MMP and TIMP family in a large series of human urothelial carcinoma samples. Laser capture microdissection is used to localise highly expressed MMP transcripts to either stromal or urothelial tissue compartments.

## MATERIALS AND METHODS

### Tissue samples and RNA preparation

In all, 169 surgical specimens of primary urothelial cell carcinoma were collected, either at cystectomy or trans-urethral resection, and snap frozen in liquid nitrogen. Twenty specimens of normal bladder urothelium were collected from areas of macroscopically normal urothelium in patients with no evidence of urothelial malignancy. Use of tissues for this study was approved by Cambridgeshire Local Research Ethics Committee (Ref 03/018). A total of 30 sections of 30 *μ*m were homogenised for RNA extraction and two 7 *μ*m ‘sandwich’ sections adjacent to tissue used for RNA extraction were sectioned, stained and assessed for cellularity and tumour grade by an independent consultant uro-histopathologist. Additionally, the sections were graded according to the degree of inflammatory cell infiltration (low, moderate and significant). Samples showing significant inflammatory cell contamination were excluded.

Total RNA was extracted using TRI Reagent™ (Sigma, Dorset, UK), following the manufacturers protocol. RNEasy Minikits™ (Qiagen, Crawley, UK), including a DNase step, were used to optimise RNA purity. Agilent 2100™ total RNA bioanalysis was performed. One microlitre of resuspended RNA from each sample was applied to an RNA 6000 NanoLabChip™, and processed according to manufacturer's instructions. All chips and reagents were sourced from Agilent Technologies™ (West Lothian, UK).

### Reverse transcription

Total RNA concentrations were determined using the Nanodrop™ ND1000 spectrophotometer (Nyxor Biotech, Paris, France). One microgram of total RNA was reverse transcribed with 2 *μ*g random hexamers (Amersham) and Superscript 11 reverse transcriptase (Invitrogen, Paisley, UK) in 20 *μ*l reactions according to the manufacturers instructions. cDNA was then diluted 1 : 100 with PCR grade water and stored at −20°C.

### Quantitative RT–PCR

For qPCR reactions, specific primers and probes for all human MMPs and TIMPs, EGF-R, VEGF-A, EMMPRIN, MET, TGF-*β* and RECK were designed as previously described (Nuttall *et al*, 2003). For 18S amplification, TaqMan Ribosomal RNA Control Reagents were purchased from Applied Biosystems, Warrington, UK. PCR reactions were performed using the ABI Prism 7700 Sequence Detection System (Applied Biosystems, Warrington, UK) following the manufacturers protocol. Reactions for 18S analyses were performed in 25 *μ*l PCR volumes containing the equivalent of 1 ng of reverse transcribed RNA, 50% TaqMan universal PCR Master Mix without UNG (Applied Biosystems, Warrington, UK), 200 nM each of the forward and reverse primers and 100 nM of probe. Amplification conditions were 2 min at 50°C, 10 min at 95°C and then 40 cycles each consisting of 15 s at 95°C and 1 min at 60°C. Reaction conditions for target gene amplification were as described above and the equivalent of 5 ng of reverse transcribed RNA was used in each reaction.

To determine relative RNA levels within the samples, standard curves for the PCR reactions were prepared from a series of two-fold dilutions of cDNA covering the range 2–0.625 ng of RNA for the 18S reaction and 20–0.5 ng of RNA for all target genes. The ABI Prism 7700 measured changes in fluorescence levels throughout the 40 cycle PCR reaction and generated a cycle threshold (*C*_T_) value for each sample correlating to the point at which amplification entered the exponential phase. This value was used as an indicator of the amount of starting template; hence a lower *C*_T_ values indicated a higher amount of initial intact cDNA.

### Laser capture microdissection

Tissue for laser capture microdissection was collected prospectively following the procedure outlined above. In all, eleven normal urothelial samples and 22 tumour samples were obtained. Five sequential sections of 7 *μ*m thickness were cut from each tissue and stained using Histogene™ staining solution (Arcturus, California, USA) following manufacturer's protocol. Slides were then immediately transferred for microdissection using a Pix Cell II laser capture microscope (Arcturus, CA, USA). This technique employs low-power infrared laser to melt a thermoplastic film over the cells of interest, to which the cells become attached.

Approximately 10 000 cells were microdissected from both stromal and epithelial/tumour compartments in each tissue. RNA was extracted using an RNEasy Micro Kit (Qiagen, Crawley, UK). Areas of tumour or stroma containing significant inflammatory cell infiltration were avoided to prevent contamination.

Total RNA was reverse transcribed and qRT–PCR performed as above. Given the low yield of RNA from such small samples, Nanodrop™ quantification was not performed, but correction for the endogenous 18S *C*_T_ value was used as an accurate measure of the amount of intact starting RNA. Transcript analysis was performed on the secreted MMPs 2, 9, 11, 13, the membrane bound MMPs 14 and 15, and TIMP-2.

To validate the accuracy of microdissection, primers and probes for Vimentin and Uroplakin were sourced and qRT–PCR performed according to manufacturer's instructions (Assays on demand, Applied Biosystems, Warrington, UK). Vimentin is primarily expressed in mesenchymally derived cells, and was used as a stromal marker. Uroplakin is a marker of urothelial differentiation and is preserved in up to 90% of epithelially derived tumours (Olsburgh *et al*, 2003).

### Statistical analysis

RNA expression levels for each target gene were normalised to the endogenous 18S rRNA levels. For grade correlation studies, two-tailed spearman's Rank Correlation was performed to determine the significance of the relationship between gene expression and increasing tumour grade. To determine the significance of differential expression in the laser captured tissue, a two-sided Mann–Whitney *U* nonparametric analysis was performed, for which a *P*-value of <0.05 was considered significant.

## RESULTS

### RNA quality control and PCR validation

After RNA assessment using the Nanodrop™ spectrophotometer, Agilent 2100™ Biolanalyser, and endogenous 18S rRNA gene, 113 tumours and 19 normal bladder specimens were considered to have intact RNA and be suitable for analysis. In all, 14 tumours were grade 1, 57 were grade 2 and 42 were grade 3. Primer and probe sets had been optimised and validated as previously described (Nuttall *et al*, 2003).

### Expression levels of MMPs and TIMPs in human bladder tissue

The PCR cycle threshold (*C*_T_) was used to quantify the expression of each gene in a given sample. Genes with *C*_T_ values of <26 cycles were considered to be very highly expressed, those with values of 26⩽30 cycles were highly expressed, 30⩽35 cycles moderately expressed and genes with *C*_T_ values in the range 35⩽40 low or absent. Table 1 shows the relative expression levels for each gene of interest in all bladder tumour samples.

In summary, MMP-2, MT1-MMP and the previously unreported MMP-28 were very highly expressed in tumour samples. MMPs 1, 7, 9, 11, 15, 19 and 23 were highly expressed while there was moderate expression of MMPs 3, 10, 12, 13, 16, 17, 24 and 25, and low expression of MMPs 8, 21, 26 and 27. No MMP-20 (enamelysin) RNA transcripts were detected in any bladder tissue. Analysis of the TIMPs confirmed very high expression of TIMP-1 and TIMP-3 and high expression of TIMP-2 and TIMP-4. EMMPRIN and VEGF-A showed a very high expression profile, with MET, TGF-*β*, and EGF-R highly expressed.

### Correlation of MMPs, TIMPs, and growth factor expression with tumour grade

**Figure 1:**
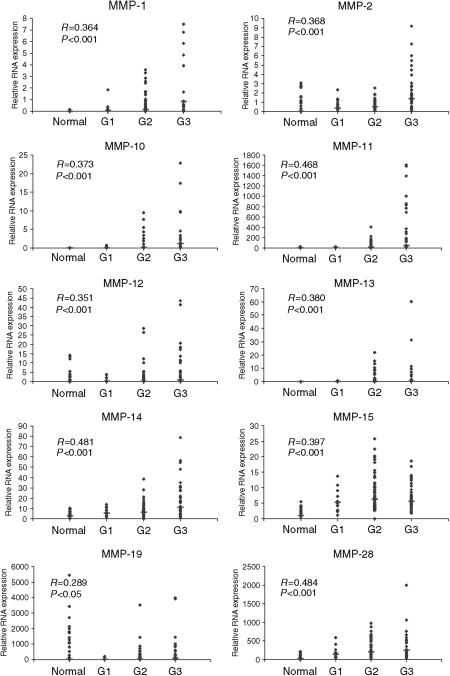
Relative mRNA expression for MMPs 1, 2, 10, 11, 12, 13, 14, 15, 19 and 28 in 19 normal tissue, 14 grade 1(G1), 57 grade 2 (G2) and 42 grade 3 (G3) tumours. RNA expression for each target gene is normalised to the endogenous 18 S value for each sample, giving a relative expression value, which allows intragene comparison. Median values are denoted by a horizontal line. Spearman Rank Correlation coefficients are shown for each gene, along with *P*-value.

**Figure 2:**
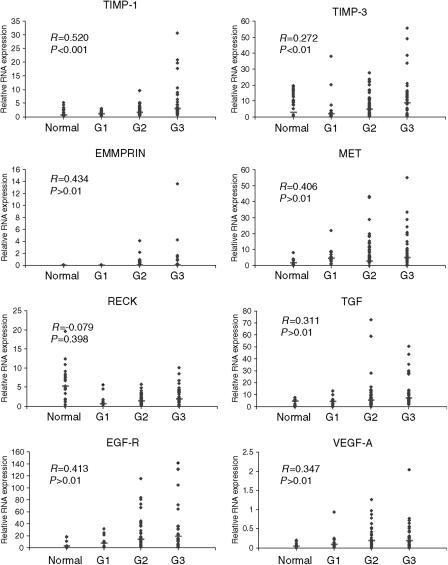
Relative RNA expression of TIMP-1, TIMP-2, EMMPRIN, MET, RECK, EGF-R, TGF*β* and VEGF-A, showing correlation with increasing tumour stage (See legend for Figure 1).

After normalisation of *C*_T_ values to individual standard curves for each gene of interest, relative RNA expression values were generated which allowed comparison of gene expression between the groups of normal tissue, low grade (grade 1), intermediate (grade 2) and high grade (grade 3) tumour tissue. The relationship between MMPs which were classified as very highly or highly expressed and tumour grade is represented in Figure 1 and that for key TIMPs, growth factors and receptors in Figure 2.

There was a significant positive correlation between transcript expression and tumour grade for MMPs 1, 2, 8, 10, 11, 12, 13, 14, 15 and 28 (*P*<0.001). At the same confidence interval, TIMP-1 and TIMP-3 also correlated with increasing tumour grade.

The growth factors and receptor genes EMMRIN, MET, TGF-*β*, VEGF-A and EGF-R all demonstrated highly significant positive correlations with tumour grade (*P*<0.001). Expression levels of the metalloproteinase inhibitor RECK were lower in all grades of tumour tissue than in normal tissue, but the relationship did not achieve statistical significance.

### Laser capture microdissection

**Figure 3:**
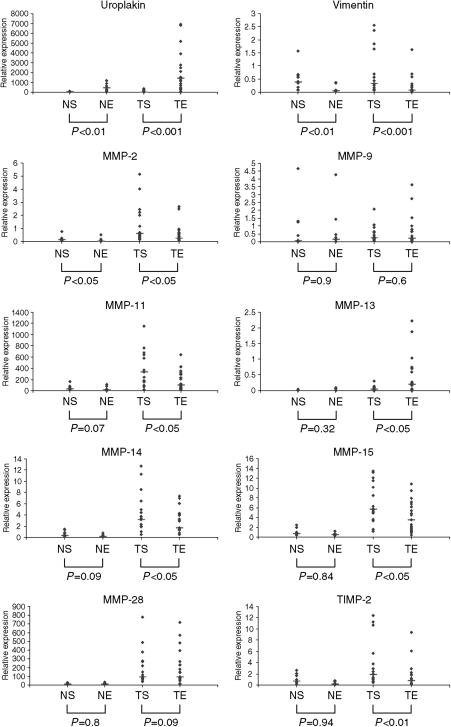
Relative RNA Expression for normal stroma (NS), normal epithelium (NE), tumour stroma (TS) and tumour epithelium (TE). Uroplakin and vimentin are epithelial and stromal validation markers respectively. Target genes are MMP-2, 9, 11, 13, 14, 15 and 28 and TIMP-2. Mann Whitney nonparametric test was used to detect significant differences between stromal and epithelial compartments. Significant differences (*P*<0.05) are highlighted in bold, and median values are denoted by a horizontal line.

The MMP genes that demonstrated both high expression and significant positive correlation with increasing tumour grade were then investigated using laser capture microdissection (LCM). RNA yields were in the region 50–80 ng for the laser capture of 10 000 cells, and RNA was well preserved, with endogenous 18S *C*_T_ values clustering closely around 15 cycles. Figure 3 summarises the results of this study.

The stromal marker gene vimentin and the epithelial marker uroplakin confirmed accurate microdissection of discrete cell populations. Vimentin expression was significantly higher in the stromal compartment in both normal tissue (*P*<0.05) and tumour tissue (*P*<0.001). Uroplakin RNA expression was significantly higher in the tumour compartment of normal tissue (*P*<0.01) and tumour tissue (*P*<0.001).

Independent of compartmental analysis, several target genes showed fold differences in expression between normal and tumour tissue which matched closely those observed in the gross profiling study. MMP-9 and MMP-28 appeared evenly distributed between stromal and epithelial compartments. In tumour tissues, MMPs 2, 11, 14, 15 and TIMP-2 were expressed at a significantly higher level in stromal tissue when compared to tissue from the epithelially derived compartment (*P*<0.05). MMP-13 was the only target gene to localise preferentially to the epithelial compartment (*P*<0.05).

## DISCUSSION

It is accepted that no single chromosomal insult, no individual protein or indeed family of proteins, can be held uniquely responsible for the transition from steady-state proliferating, regenerating epithelial cells to a high grade, invasive tumour phenotype. The process is complex, requiring a temporal or spatial combination of tumourigenic events. Matrix metalloproteinases, by virtue of their diverse spectrum of activity, are likely to play key roles in many of these biological processes.

MMPs have been implicated in angiogenesis, either directly or via the upregulation or activation of pro-angiogenic factors such as VEGF (Bergers *et al*, 2000; Sounni *et al*, 2002) and in the case of MT1-MMP there is recent evidence that this regulation may occur at a transcriptional level (Sounni *et al*, 2004). However, not all MMP activity is proangiogenic; for example the gelatinases and MMP-7 can cleave plasminogen to form the angiogenesis inhibitor angiostatin (Cornelius *et al*, 1998).

MMPs 7 and 11 are involved in apoptotic regulation through several pathways including FAS death receptor ligand cleavage and HB-EGF activation (Mitsiades *et al*, 2001; Wu *et al*, 2001; Yu *et al*, 2002). Additionally, MMPs cleave cell adhesion molecules such as E cadherin (Lochter *et al*, 1997), and modulate the immune response to tumours through chemokine deactivation (McQuibban *et al*, 2000, 2001, 2002) and T-lymphocyte suppression (Sheu *et al*, 2001).

This work represents the first comprehensive quantitative RNA transcript profile of the entire MMP and TIMP family in a large cohort of human bladder tumours. Quantitative RT–PCR is accurate, with high specificity and sensitivity, registering the equivalent of <1 mRNA transcript per cell. The robust nature of this technique is evidenced within this study. We observed similar fold differences in MMP expression between normal and tumour tissue in both the 132 gross profiling samples and the 31 tissue sections used for microdissection. These two tissue cohorts were processed identically but at different times, and originated from two entirely different patient populations.

This analysis confirms the importance of well-documented MMPs, but also highlights elevated expression and correlation with increasing tumour grade for novel genes, which represent interesting targets for further analysis. In general, we observed that low-grade (G1) tumours show expression patterns similar to normal tissue, independent of tumour stage. If analysis is restricted to non-invasive stage pTa/pT1 tumours, for all MMPs, the correlation with increasing grade remains significant (data not shown).

In urothelial cell carcinoma, in common with most disease processes, the most widely documented MMPs are the gelatinases (MMPs 2 and 9), which have been shown to correlate with increasing tumour grade using techniques such as quantitative zymography (Davies *et al*, 1993; Kanda *et al*, 2000). MMP-2 expression was significantly higher in muscle invasive disease when analysed by semi quantitative RT–PCR (Kanayama *et al*, 1998; Xu *et al*, 2002) and immunohistochemistry (Sumi *et al*, 2003). In this study, MMP-2 was confirmed as being very highly expressed in tumour tissue, and to correlate significantly with increasing tumour grade. MMP-2 upregulation may well represent an exaggerated host response, as LCM analysis confirmed that RNA transcripts are located primarily in the host stroma rather than epithelially derived tumour cells. It is likely that RNA transcript localisation accurately reflect protein expression, as similar distributions have been noted in bladder with immunohistochemistry (Sumi *et al*, 2003).

In a series of 51 superficial bladder cancers, MMP-9 RNA expression was 2.5 fold higher in the tumours of patients with subsequent recurrence (Hara *et al*, 2001); we have shown that, while highly expressed in tumour tissue, MMP-9 just failed to demonstrate a significant correlation with tumour grade or to localise to a specific tumour compartment. This may reflect the complex balance between pro- and antitumourigenic activity of several MMPs and their endogenous inhibitors (Egeblad and Werb, 2002).

There has been, until now, limited evidence underlining the importance of other MMPs in bladder cancer. The concentration of MMP-1 in urine correlates with tumour stage and grade (Nutt *et al*, 1998; Durkan *et al*, 2001), a relationship confirmed in tissue samples in our study. Using immunohistochemistry, Nakopoulou *et al* (2001) showed MMP-1 and MMP-3 to be overexpressed in bladder tumour tissue, with only the former showing a positive correlation with either stage or grade. Our profiling verified that MMP3 was only moderately expressed in normal and tumour tissue, with no relationship with increasing grade. Other proteinases, such as MMP-7, 11 and 13 have been detected in increased levels in bladder cancers, but have in general failed to demonstrate statistically significant positive relationships with pathological end points (Bostrom *et al*, 2000; Mueller *et al*, 2000; Sumi *et al*, 2003). We have demonstrated that the secreted MMPs 10–13 inclusive all show significant positive correlation with tumour grade. Microdissection revealed that MMP-11 was located primarily in the host stroma; in concordance, Mueller *et al* (2000) localised MMP-11 protein to peritumour fibroblasts in breast and bladder cancer. However, MMP-13 was the only proteinase in our study to localise to the epithelially derived tumour component. Using *in situ* hybridisation and immunohistochemistry, Bostrom *et al* (2000) demonstrated MMP-13 RNA and protein localisation to the cells at the leading edge of invading bladder tumours, a finding confirmed in oesophageal tumours by Etoh *et al* (2000).

Interestingly MMP-28, or epilysin, is very highly expressed in bladder samples, especially in high-grade tumours. This protein has been documented to be elevated in a number of human malignancies including colonic adenocarcinoma and ovarian carcinoma using nonquantitative PCR (Marchenko and Strongin, 2001). However, in contrast to these findings, Bister *et al* (2004) have suggested that MMP-28 expression is reduced in colonic cancer epithelium, when compared to normal tissue. To our knowledge, MMP-28 has not previously been investigated in bladder cancer.

Membrane bound MMPs are of increasing interest, although the extent of their investigation in urothelial cancers is limited. MT1-MMP (MMP-14) and MT2-MMP (MMP-15) both demonstrated an extremely highly significant RNA expression profile in this study, and localised to the tumour stroma. They have been shown to be of interest in breast carcinoma with the former showing a distribution similar to MMP-2, and to correlate with increasing tumour stage (Ueno *et al*, 1997). Elevated expression of MT1-MMP has been reported in a variety of solid tumours including cervix (Gilles *et al*, 1996), head and neck and colon (Okada *et al*, 1995). In tissues from 41 patients with bladder cancer, using nonquantitative RT–PCR, MT1-MMP was shown to associate strongly with decreased survival (Kanayama *et al*, 1998). Nakahara *et al* (1997) observed the preferential localisation and functional significance of MT1-MMP in the invadopodia of melanoma cells. Localisation studies suggest that stromally derived MT1-MMP may be cleaved from the cell surface and that the resulting soluble protein is endocytosed and re-expressed by the tumour cells (Chenard *et al*, 1999), but this is currently unsubstantiated.

The endogenous TIMPs have a complex relationship with the MMP family. While all four TIMPs inhibit most MMPs (with the exception of TIMP-1 which has a limited inhibitory effect on the membrane bound MMPs), they are also implicated in protumourigenic activities including promotion of cell proliferation, apoptotic resistance and, in the case of TIMP-2, the activation of latent MMP-2 in a complex with MT1-MMP (Baker *et al*, 2002). We found TIMP-1, TIMP-2 and TIMP-3 to be highly expressed both in normal tissue and in tumour tissue, with increased expression in high-grade tumours. Similar patterns have been reported in bladder, colorectal, breast and lung cancer (Grignon *et al*, 1996; Kossakowska *et al*, 1996). Several reports have postulated the MMP/TIMP ratio as significant in predicting either tumour recurrence or progression in bladder cancer, concentrating on MMPs 1, 2 and 9 and TIMPs 1 and 2 (Gohji *et al*, 1996; Durkan *et al*, 2003). A rise in MMP/TIMP ratio may indicate a net increase in proteolytic activity. Analysis of our profiling data shows an increase in MMP/TIMP ratios for combinations of all the above MMPs and TIMPs in high-grade tumours, suggesting a markedly proteolytic microenvironment.

We also profiled mRNA transcript expression of several growth factors and receptors implicated both in the upstream regulation and downstream signalling pathways of the MMPs. The endogenous MMP inducer EMMPRIN stimulates stromal fibroblasts to produce MMPs 1, 2 and 3 (Caudroy *et al*, 2002). It was expressed in high levels in high-grade tumours in this study. Conversely, the membrane anchored MMP inhibitor RECK (reversion inducing cysteine-rich protein with kazal motifs) has been shown by Takeuchi *et al* (2004) to correlate inversely with recurrence and microvessel formation in colorectal cancer.

We confirmed that VEGF-A, TGF*β*, EGF-R and c-MET were all highly expressed in tumour tissue, and correlated positively with tumour grade. VEGF-A is upregulated by MT1-MMP promoting angiogenesis, as discussed previously and TGF-*β* is a substrate of the gelatinases. Once activated, it is involved in endothelial proliferation and tubulogenesis (Stamenkovic, 2000). Evidence for the importance of EGF-R in progression of epithelial tumours including bladder cancer exits both *in vivo* and *in vitro* (Popov *et al*, 2004). It has been shown to be highly expressed in high grade bladder tumours using immunohistochemistry (Neal *et al*, 1990). The Hepatocyte Growth Factor (HGF) receptor c-MET tyrosine kinase is expressed on epithelially derived tumour cells, and is involved in HGF mediated activation of both MMP2 and MT1-MMP (Hamasuna *et al*, 1999).

Large-scale quantitative mRNA transcript profiling combined with laser capture microdissection of specific tumour compartments generates a powerful ‘snapshot view’ of gene expression in normal and tumour tissues. While no true functional conclusions can be drawn as to the roles of the MMPs TIMPs, growth factors or receptors profiled, the upregulation of such a broad spectrum of the MMP family suggests they are an integral part of the complex pathways involved in bladder tumour progression. Studies have demonstrated upregulation of multiple members of the MMP family in other tumour types including prostate and glioblastoma, but the expression patterns vary significantly between tissue types (Nuttall *et al*, 2003; Riddick *et al*, 2005). In a recent study by Martinez *et al* (2005), a wide spectrum of MMPs were found to be upregulated in early murine colonic adenomas; those MMPs located on chromosome 9 showed significantly greater upregulation that those expressed on other chromosomes, suggesting a shared regulatory mechanism resulting in coordinated deregulation of expression in tumour tissue. Although the majority of the chromosome 9 MMPs were shown to be upregulated in our study, several, including MMPs 3, 8 and 10, demonstrated only moderate or low expression, leaving the concept of chromosomal based coregulation of MMP expression open to further debate.

Our work confirms the importance of well-documented genes, such as MMP-2 and MT1-MMP, but also highlights several new proteases including MMP-28, whose localisation and function in early bladder cancer is under-investigated. Further protein based and functional research will help to characterise the roles of these newer proteinases and underline their potential as prognostic, diagnostic or therapeutic markers in early bladder cancer.

## Figures and Tables

**Table 1:** Relative RNA transcript expression (expressed as mean *C*_T_ value) for all MMPs, TIMPs, EMMPRIN, MET, RECK, VEGF-A, TGF and EGF-R in bladder tumour tissue (*n*=113)

**Target gene**	**Expression level**	**Target gene**	**Expression level**
MMP-1	28.2	MMP-23	29.8
MMP-2	22.5	MMP-24	33.2
MMP-3	31.3	MMP-25	33.2
MMP-7	27.6	MMP-26	38.7
MMP-8	38.5	MMP-27	36.2
MMP-9	29.2	MMP-28	26.5
MMP-10	30.2	TIMP-1	22.5
MMP-11	27	TIMP-2	28.2
MMP-12	30.4	TIMP-3	23.5
MMP-13	31.1	TIMP-4	31.5
MMP-14	24.7		
MMP-15	26.6	EMMPRIN	25.8
MMP-16	32.9	MET	27.8
MMP-17	33.4	RECK	30.4
MMP-19	28.5	VEGF-A	23.9
MMP-20	40	TGF	28.8
MMP-21	35.8	EGF-R	26.3

	**Expression level**	**Mean *C*_T_ value**	
	Low	35<39	
	Moderate	30<35	
	High	26<30	
	Very high	<26	

Genes with *C*_T_ values of ⩽25 cycles were considered to be very highly expressed, those with values of 26⩽30 cycles were highly expressed, 30⩽35 cycles moderately expressed and genes with *C*_T_ values in the range 35⩽40 low or absent.

## References

[bib1] Baker AH, Edwards DR, Murphy G (2002) Metalloproteinase inhibitors: biological actions and therapeutic opportunities. J Cell Sci 115: 3719–3727 12235282 10.1242/jcs.00063

[bib2] Bergers G, Brekken R, McMahon G, Vu TH, Itoh T, Tamaki K, Tanzawa K, Thorpe P, Itohara S, Werb Z, Hanahan D (2000) Matrix metalloproteinase-9 triggers the angiogenic switch during carcinogenesis. Nat Cell Biol 2: 737–744 11025665 10.1038/35036374 PMC2852586

[bib3] Birkedal-Hansen H, Moore WG, Bodden MK, Windsor LJ, Birkedal-Hansen B, DeCarlo A, Engler JA (1993) Matrix metalloproteinases: a review. Crit Rev Oral Biol Med 4: 197–250 8435466 10.1177/10454411930040020401

[bib4] Bister VO, Salmela MT, Karjalainen-Lindsberg ML, Uria J, Lohi J, Puolakkainen P, Lopez-Otin C, Saarialho-Kere U (2004) Differential expression of three matrix metalloproteinases, MMP-19, MMP-26, and MMP-28, in normal and inflamed intestine and colon cancer. Dig Dis Sci 49: 653–661 15185874 10.1023/b:ddas.0000026314.12474.17

[bib5] Bostrom PJ, Ravanti L, Reunanen N, Aaltonen V, Soderstrom KO, Kahari VM, Laato M (2000) Expression of collagenase-3 (matrix metalloproteinase-13) in transitional-cell carcinoma of the urinary bladder. Int J Cancer 88: 417–423 11054671

[bib6] Caudroy S, Polette M, Nawrocki-Raby B, Cao J, Toole BP, Zucker S, Birembaut P (2002) EMMPRIN-mediated MMP regulation in tumor and endothelial cells. Clin Exp Metastasis 19: 697–702 12553375 10.1023/a:1021350718226

[bib7] Chang C, Werb Z (2001) The many faces of metalloproteases: cell growth, invasion, angiogenesis and metastasis. Trends Cell Biol 11: S37–S43 11684441 10.1016/s0962-8924(01)02122-5 PMC2788992

[bib8] Chenard MP, Lutz Y, Mechine-Neuville A, Stoll I, Bellocq JP, Rio MC, Basset P (1999) Presence of high levels of MT1-MMP protein in fibroblastic cells of human invasive carcinomas. Int J Cancer 82: 208–212 10389754 10.1002/(sici)1097-0215(19990719)82:2<208::aid-ijc10>3.0.co;2-9

[bib9] Cornelius LA, Nehring LC, Harding E, Bolanowski M, Welgus HG, Kobayashi DK, Pierce RA, Shapiro SD (1998) Matrix metalloproteinases generate angiostatin: effects on neovascularization. J Immunol 161: 6845–6852 9862716

[bib10] Coussens LM, Fingleton B, Matrisian LM (2002) Matrix metalloproteinase inhibitors and cancer: trials and tribulations. Science 295: 2387–2392 11923519 10.1126/science.1067100

[bib11] Davies B, Waxman J, Wasan H, Abel P, Williams G, Krausz T, Neal D, Thomas D, Hanby A, Balkwill F (1993) Levels of matrix metalloproteases in bladder cancer correlate with tumor grade and invasion. Cancer Res 53: 5365–5369 8221672

[bib12] Durkan GC, Nutt JE, Marsh C, Rajjayabun PH, Robinson MC, Neal DE, Lunec J, Mellon JK (2003) Alteration in urinary matrix metalloproteinase-9 to tissue inhibitor of metalloproteinase-1 ratio predicts recurrence in nonmuscle-invasive bladder cancer. Clin Cancer Res 9: 2576–2582 12855633

[bib13] Durkan GC, Nutt JE, Rajjayabun PH, Neal DE, Lunec J, Mellon JK (2001) Prognostic significance of matrix metalloproteinase-1 and tissue inhibitor of metalloproteinase-1 in voided urine samples from patients with transitional cell carcinoma of the bladder. Clin Cancer Res 7: 3450–3456 11705862

[bib14] Egeblad M, Werb Z (2002) New functions for the matrix metalloproteinases in cancer progression. Nat Rev Cancer 2: 161–174 11990853 10.1038/nrc745

[bib15] Etoh T, Inoue H, Yoshikawa Y, Barnard GF, Kitano S, Mori M (2000) Increased expression of collagenase-3 (MMP-13) and MT1-MMP in oesophageal cancer is related to cancer aggressiveness. Gut 47: 50–56 10861264 10.1136/gut.47.1.50 PMC1727967

[bib16] Gilles C, Polette M, Piette J, Munaut C, Thompson EW, Birembaut P, Foidart JM (1996) High level of MT-MMP expression is associated with invasiveness of cervical cancer cells. Int J Cancer 65: 209–213 8567119 10.1002/(SICI)1097-0215(19960117)65:2<209::AID-IJC14>3.0.CO;2-8

[bib17] Gohji K, Fujimoto N, Fujii A, Komiyama T, Okawa J, Nakajima M (1996) Prognostic significance of circulating matrix metalloproteinase-2 to tissue inhibitor of metalloproteinases-2 ratio in recurrence of urothelial cancer after complete resection. Cancer Res 56: 3196–3198 8764105

[bib18] Grignon DJ, Sakr W, Toth M, Ravery V, Angulo J, Shamsa F, Pontes JE, Crissman JC, Fridman R (1996) High levels of tissue inhibitor of metalloproteinase-2 (TIMP-2) expression are associated with poor outcome in invasive bladder cancer. Cancer Res 56: 1654–1659 8603416

[bib19] Hamasuna R, Kataoka H, Moriyama T, Itoh H, Seiki M, Koono M (1999) Regulation of matrix metalloproteinase-2 (MMP-2) by hepatocyte growth factor/scatter factor (HGF/SF) in human glioma cells: HGF/SF enhances MMP-2 expression and activation accompanying up-regulation of membrane type-1 MMP. Int J Cancer 82: 274–281 10389763 10.1002/(sici)1097-0215(19990719)82:2<274::aid-ijc19>3.0.co;2-2

[bib20] Hara I, Miyake H, Hara S, Arakawa S, Kamidono S (2001) Significance of matrix metalloproteinases and tissue inhibitors of metalloproteinase expression in the recurrence of superficial transitional cell carcinoma of the bladder. J Urol 165: 1769–1772 11342973

[bib21] Jemal A, Tiwari RC, Murray T, Ghafoor A, Samuels A, Ward E, Feuer EJ, Thun MJ (2004) Cancer statistics, 2004. CA Cancer J Clin 54: 8–29 14974761 10.3322/canjclin.54.1.8

[bib22] Kanayama H, Yokota K, Kurokawa Y, Murakami Y, Nishitani M, Kagawa S (1998) Prognostic values of matrix metalloproteinase-2 and tissue inhibitor of metalloproteinase-2 expression in bladder cancer. Cancer 82: 1359–1366 9529029

[bib23] Kanda K, Takahashi M, Murakami Y, Kanayama H, Kagawa S (2000) The role of the activated form of matrix metalloproteinase-2 in urothelial cancer. BJU Int 86: 553–557 10971291 10.1046/j.1464-410x.2000.00734.x

[bib24] Knauper V, Cowell S, Smith B, Lopez-Otin C, O'Shea M, Morris H, Zardi L, Murphy G (1997) The role of the C-terminal domain of human collagenase-3 (MMP-13) in the activation of procollagenase-3, substrate specificity, and tissue inhibitor of metalloproteinase interaction. J Biol Chem 272: 7608–7616 9065415 10.1074/jbc.272.12.7608

[bib25] Kossakowska AE, Huchcroft SA, Urbanski SJ, Edwards DR (1996) Comparative analysis of the expression patterns of metalloproteinases and their inhibitors in breast neoplasia, sporadic colorectal neoplasia, pulmonary carcinomas and malignant non-Hodgkin's lymphomas in humans. Br J Cancer 73: 1401–1408 8645587 10.1038/bjc.1996.266 PMC2074489

[bib26] Lochter A, Galosy S, Muschler J, Freedman N, Werb Z, Bissell MJ (1997) Matrix metalloproteinase stromelysin-1 triggers a cascade of molecular alterations that leads to stable epithelial-to-mesenchymal conversion and a premalignant phenotype in mammary epithelial cells. J Cell Biol 139: 1861–1872 9412478 10.1083/jcb.139.7.1861 PMC2132651

[bib27] Lohi J, Wilson CL, Roby JD, Parks WC (2001) Epilysin, a novel human matrix metalloproteinase (MMP-28) expressed in testis and keratinocytes and in response to injury. J Biol Chem 276: 10134–10144 11121398 10.1074/jbc.M001599200

[bib28] Lutzeyer W, Rubben H, Dahm H (1982) Prognostic parameters in superficial bladder cancer: an analysis of 315 cases. J Urol 127: 250–252 7062375 10.1016/s0022-5347(17)53725-8

[bib29] Marchenko GN, Strongin AY (2001) MMP-28, a new human matrix metalloproteinase with an unusual cysteine-switch sequence is widely expressed in tumors. Gene 265: 87–93 11255011 10.1016/s0378-1119(01)00360-2

[bib30] Martinez C, Bhattacharya S, Freeman T, Churchman M, Ilyas M (2005) Expression profiling of murine intestinal adenomas reveals early deregulation of multiple matrix metalloproteinase (Mmp) genes. J Pathol 206: 100–110 15809971 10.1002/path.1755

[bib31] McQuibban GA, Butler GS, Gong JH, Bendall L, Power C, Clark-Lewis I, Overall CM (2001) Matrix metalloproteinase activity inactivates the CXC chemokine stromal cell-derived factor-1. J Biol Chem 276: 43503–43508 11571304 10.1074/jbc.M107736200

[bib32] McQuibban GA, Gong JH, Tam EM, McCulloch CA, Clark-Lewis I, Overall CM (2000) Inflammation dampened by gelatinase A cleavage of monocyte chemoattractant protein-3. Science 289: 1202–1206 10947989 10.1126/science.289.5482.1202

[bib33] McQuibban GA, Gong JH, Wong JP, Wallace JL, Clark-Lewis I, Overall CM (2002) Matrix metalloproteinase processing of monocyte chemoattractant proteins generates CC chemokine receptor antagonists with anti-inflammatory properties *in vivo*. Blood 100: 1160–1167 12149192

[bib34] Mitsiades N, Yu WH, Poulaki V, Tsokos M, Stamenkovic I (2001) Matrix metalloproteinase-7-mediated cleavage of Fas ligand protects tumor cells from chemotherapeutic drug cytotoxicity. Cancer Res 61: 577–581 11212252

[bib35] Mueller J, Steiner C, Hofler H (2000) Stromelysin-3 expression in noninvasive and invasive neoplasms of the urinary bladder. Hum Pathol 31: 860–865 10923925 10.1053/hupa.2000.8447

[bib36] Murphy G, Knauper V, Cowell S, Hembry R, Stanton H, Butler G, Freije J, Pendas AM, Lopez-Otin C (1999) Evaluation of some newer matrix metalloproteinases. Ann NY Acad Sci 878: 25–39 10415718 10.1111/j.1749-6632.1999.tb07672.x

[bib37] Nakahara H, Howard L, Thompson EW, Sato H, Seiki M, Yeh Y, Chen WT (1997) Transmembrane/cytoplasmic domain-mediated membrane type 1-matrix metalloprotease docking to invadopodia is required for cell invasion. Proc Natl Acad Sci USA 94: 7959–7964 9223295 10.1073/pnas.94.15.7959 PMC21537

[bib38] Nakopoulou L, Gakiopoulou H, Zervas A, Giannopoulou I, Constantinides C, Lazaris AC, Liapis H, Kyriakou G, Dimopoulos C (2001) MMP-3 mRNA and MMP-3 and MMP-1 proteins in bladder cancer: a comparison with clinicopathologic features and survival. Appl Immunohistochem Mol Morphol 9: 130–137 11396630 10.1097/00129039-200106000-00005

[bib39] Neal DE, Sharples L, Smith K, Fennelly J, Hall RR, Harris AL (1990) The epidermal growth factor receptor and the prognosis of bladder cancer. Cancer 65: 1619–1625 2311071 10.1002/1097-0142(19900401)65:7<1619::aid-cncr2820650728>3.0.co;2-q

[bib40] Nutt JE, Mellon JK, Qureshi K, Lunec J (1998) Matrix metalloproteinase-1 is induced by epidermal growth factor in human bladder tumour cell lines and is detectable in urine of patients with bladder tumours. Br J Cancer 78: 215–220 9683296 10.1038/bjc.1998.467 PMC2062898

[bib41] Nuttall RK, Pennington CJ, Taplin J, Wheal A, Yong VW, Forsyth PA, Edwards DR (2003) Elevated membrane-type matrix metalloproteinases in gliomas revealed by profiling proteases and inhibitors in human cancer cells. Mol Cancer Res 1: 333–345 12651907

[bib42] Okada A, Bellocq JP, Rouyer N, Chenard MP, Rio MC, Chambon P, Basset P (1995) Membrane-type matrix metalloproteinase (MT-MMP) gene is expressed in stromal cells of human colon, breast, and head and neck carcinomas. Proc Natl Acad Sci USA 92: 2730–2734 7708715 10.1073/pnas.92.7.2730 PMC42292

[bib43] Olsburgh J, Harnden P, Weeks R, Smith B, Joyce A, Hall G, Poulsom R, Selby P, Southgate J (2003) Uroplakin gene expression in normal human tissues and locally advanced bladder cancer. J Pathol 199: 41–49 12474225 10.1002/path.1252

[bib44] Popov Z, Gil-Diez-De-Medina S, Ravery V, Hoznek A, Bastuji-Garin S, Lefrere-Belda MA, Abbou CC, Chopin DK (2004) Prognostic value of EGF receptor and tumor cell proliferation in bladder cancer: therapeutic implications. Urol Oncol 22: 93–101 15082004 10.1016/j.urolonc.2004.01.001

[bib45] Raghavan D, Shipley WU, Garnick MB, Russell PJ, Richie JP (1990) Biology and management of bladder cancer. N Engl J Med 322: 1129–1138 2181313 10.1056/NEJM199004193221607

[bib46] Riddick AC, Shukla CJ, Pennington CJ, Bass R, Nuttall RK, Hogan A, Sethia KK, Ellis V, Collins AT, Maitland NJ, Ball RY, Edwards DR (2005) Identification of degradome components associated with prostate cancer progression by expression analysis of human prostatic tissues. Br J Cancer 92: 2171–2180 15928670 10.1038/sj.bjc.6602630 PMC2361819

[bib47] Seiki M (2002) The cell surface: the stage for matrix metalloproteinase regulation of migration. Curr Opin Cell Biol 14: 624–632 12231359 10.1016/s0955-0674(02)00363-0

[bib48] Sheu BC, Hsu SM, Ho HN, Lien HC, Huang SC, Lin RH (2001) A novel role of metalloproteinase in cancer-mediated immunosuppression. Cancer Res 61: 237–242 11196168

[bib49] Sounni NE, Devy L, Hajitou A, Frankenne F, Munaut C, Gilles C, Deroanne C, Thompson EW, Foidart JM, Noel A (2002) MT1-MMP expression promotes tumor growth and angiogenesis through an up-regulation of vascular endothelial growth factor expression. FASEB J 16: 555–564 11919158 10.1096/fj.01-0790com

[bib50] Sounni NE, Roghi C, Chabottaux V, Janssen M, Munaut C, Maquoi E, Galvez BG, Gilles C, Frankenne F, Murphy G, Foidart JM, Noel A (2004) Up-regulation of vascular endothelial growth factor-A by active membrane-type 1 matrix metalloproteinase through activation of Src-tyrosine kinases. J Biol Chem 279: 13564–13574 14729679 10.1074/jbc.M307688200

[bib51] Stamenkovic I (2000) Matrix metalloproteinases in tumor invasion and metastasis. Semin Cancer Biol 10: 415–433 11170864 10.1006/scbi.2000.0379

[bib52] Sternlicht MD, Werb Z (2001) How matrix metalloproteinases regulate cell behavior. Annu Rev Cell Dev Biol 17: 463–516 11687497 10.1146/annurev.cellbio.17.1.463 PMC2792593

[bib53] Sumi T, Yoshida H, Hyun Y, Yasui T, Matsumoto Y, Hattori K, Sugimura K, Kawashima H, Nakatani T, Ishiko O (2003) Expression of matrix metalloproteinases in human transitional cell carcinoma of the urinary bladder. Oncol Rep 10: 345–349 12579270

[bib54] Takeuchi T, Hisanaga M, Nagao M, Ikeda N, Fujii H, Koyama F, Mukogawa T, Matsumoto H, Kondo S, Takahashi C, Noda M, Nakajima Y (2004) The membrane-anchored matrix metalloproteinase (MMP) regulator RECK in combination with MMP-9 serves as an informative prognostic indicator for colorectal cancer. Clin Cancer Res 10: 5572–5579 15328199 10.1158/1078-0432.CCR-03-0656

[bib55] Torti FM, Lum BL (1984) The biology and treatment of superficial bladder cancer. J Clin Oncol 2: 505–531 6427417 10.1200/JCO.1984.2.5.505

[bib56] Ueno H, Nakamura H, Inoue M, Imai K, Noguchi M, Sato H, Seiki M, Okada Y (1997) Expression and tissue localization of membrane-types 1, 2, and 3 matrix metalloproteinases in human invasive breast carcinomas. Cancer Res 57: 2055–2060 9158005

[bib57] Vihinen P, Kahari VM (2002) Matrix metalloproteinases in cancer: prognostic markers and therapeutic targets. Int J Cancer 99: 157–166 11979428 10.1002/ijc.10329

[bib58] Wu E, Mari BP, Wang F, Anderson IC, Sunday ME, Shipp MA (2001) Stromelysin-3 suppresses tumor cell apoptosis in a murine model. J Cell Biochem 82: 549–555 11500932 10.1002/jcb.1181

[bib59] Xu K, Hou S, Du Z (2002) Prognostic value of matrix metalloproteinase-2 and tissue inhibitor of metalloproteinase-2 in bladder carcinoma. Chin Med J (Engl) 115: 743–745 12133547

[bib60] Yu WH, Woessner Jr JF, McNeish JD, Stamenkovic I (2002) CD44 anchors the assembly of matrilysin/MMP-7 with heparin-binding epidermal growth factor precursor and ErbB4 and regulates female reproductive organ remodeling. Genes Dev 16: 307–323 11825873 10.1101/gad.925702 PMC155329

